# Phenotypic diploidization in plant functional traits uncovered by synthetic neopolyploids in *Dianthus broteri*

**DOI:** 10.1093/jxb/erab179

**Published:** 2021-04-28

**Authors:** Juan José Domínguez-Delgado, Javier López-Jurado, Enrique Mateos-Naranjo, Francisco Balao

**Affiliations:** Departamento de Biología Vegetal y Ecología, Facultad de Biología, Universidad de Sevilla, Apdo. 1095, 41080-Sevilla, Spain; Departamento de Biología Vegetal y Ecología, Facultad de Biología, Universidad de Sevilla, Apdo. 1095, 41080-Sevilla, Spain; Departamento de Biología Vegetal y Ecología, Facultad de Biología, Universidad de Sevilla, Apdo. 1095, 41080-Sevilla, Spain; Departamento de Biología Vegetal y Ecología, Facultad de Biología, Universidad de Sevilla, Apdo. 1095, 41080-Sevilla, Spain; CSIRO Agriculture and Food, Australia

**Keywords:** Chlorophyll fluorescence, colchicine-induced autopolyploidy, *Dianthus broteri*, functional traits, leaf gas exchange, phenotypic diploidization, photosynthesis

## Abstract

Whole-genome duplication and post-polyploidization genome downsizing play key roles in the evolution of land plants; however, the impact of genomic diploidization on functional traits still remains poorly understood. Using *Dianthus broteri* as a model, we compared the ecophysiological behaviour of colchicine-induced neotetraploids (4*x*_Neo_) to diploids (2*x*) and naturally occurring tetraploids (4*x*_Nat_). Leaf gas-exchange and chlorophyll fluorescence analyses were performed in order to asses to what extent post-polyploidization evolutionary processes have affected 4*x*_Nat_. Genomic diploidization and phenotypic novelty were evident. Distinct patterns of variation revealed that post-polyploidization processes altered the phenotypic shifts directly mediated by genome doubling. The photosynthetic phenotype was affected in several ways but the main effect was phenotypic diploidization (i.e. 2*x* and 4*x*_Nat_ were closer to each other than to 4*x*_Neo_). Overall, our results show the potential benefits of considering experimentally synthetized versus naturally established polyploids when exploring the role of polyploidization in promoting functional divergence.

## Teaser

Colchicine-induced polyploidization in *Dianthus broteri* demonstrates that ‘phenotypic diploidization’ occurs in functional traits, which can lead to the creative role of polyploidization being underestimated.

## Introduction

Polyploidy, the genomic condition resulting from whole-genome duplication (WGD), is a widespread phenomenon that is considered to be a major driving force in evolution and diversification of flowering plants (Chen, 2007; Soltis *et al.*, 2009; Tank *et al.*, 2015; Landis *et al.*, 2018). Although WGD is usually accompanied by hybridization (i.e. allopolyploidy), pure WGD (i.e. autopolyploidy) *per se* can induce immediate changes at the cellular and phenotypic levels as a result of the dramatic changing of the relationship between gene copy number and other cellular components (Doyle and Coate, 2019). Newly formed polyploids (hereafter referred to as neopolyploids) usually show an increase of cell size in concert with the DNA content (‘nucleotypic effect’; Stebbins, 1971) and hence produce bigger organs (te Beest *et al.*, 2012). In addition, changes in cell volume associated with WGD may promote phenotypic novelty (Soltis *et al.*, 2014; Wendel, 2015). The most common phenotypic changes in neopolyploid plants include larger stomatal size, thicker leaf morphology, and more chloroplasts within each guard cell, which can modify physiological traits such as transpiration, and photosynthetic and growth rates (Masterson, 1994; Levin, 2002; Maherali *et al.*, 2009; Liu *et al.*, 2011).

Following WGD, polyploids start an adaptive process that can be divided into two evolutionary time-frames (Dodsworth *et al.*, 2016). In the short term, neopolyploids need to survive and to establish by overcoming several intrinsic disadvantages (such as ‘genomic shock’ and aberrant meiosis; Comai, 2005), as well as extrinsic ones (i.e. minority cytotype exclusion, a frequency-dependent mating disadvantage; Levin, 1975). In this initial stage, polyploids need to outcompete their parents or ecologically differentiate from them. Hence, the adaptive shifts required for the establishment of neopolyploids (Soltis *et al.*, 2014; Glick *et al.*, 2016) usually bring them to inhabit new niches, especially under unstable environmental conditions (te Beest *et al.*, 2012; López-Jurado *et al.*, 2020). Polyploidy-mediated changes in physiological and ecological tolerances can enhance selective advantages and lead to the initial short-term survival and establishment of polyploids (Pandit *et al.*, 2011; Soltis *et al.*, 2014; Münzbergová and Haisel, 2019; Rice *et al.*, 2019). In spite of the importance of ecophysiological aspects of niche divergence in autopolyploids, actual supporting evidence is scarce and is usually focused on drought tolerance (Maherali *et al.*, 2009; Soltis *et al.*, 2010; Liu *et al.*, 2011; Spoelhof *et al.*, 2017). Furthermore, the role of photosynthetic-related shifts in the establishment of polyploids has largely been overlooked (but see Vyas *et al.*, 2007 and López-Jurado *et al.*, 2020).

Once a polyploid has been effectively established, a longer-term process of evolution begins as it becomes a quasi-diploid, through the diploidization process. The most common phenomenon during diploidization is genome downsizing caused by the loss of duplicate genes (fractionation) and small genomic fragments (Cheng *et al.*, 2018), which might be noticeable when comparing the monoploid genome size (1Cx) among different ploidy levels (Balao *et al.*, 2009). In addition, there are other evolutionary processes that act in parallel and shape the genomic, transcriptomic, and phenotypic structure of the mesopolyploids to allow their functional diploidization, such as gene subfunctionalization and neofunctionalization, gene-silencing, activation of transposable elements, or genome rearrangements (Dodsworth *et al.*, 2016).

Notwithstanding all these well-known effects of WGD at the genomic, morphological, anatomical, physiological, and ecological scales (Ramsey and Schemske, 2002; Madlung *et al.*, 2005; Soltis *et al.*, 2014; Spoelhof *et al.*, 2017), discerning the immediate impact of WGD from the short-term and long-term adaptive effects is challenging. Most previous studies have been conducted in naturally occurring polyploid complexes (Thompson and Merg, 2008; Balao *et al.*, 2011; Manzaneda *et al.*, 2012; Pegoraro *et al.*, 2019) whose lineages have already experienced selection and diploidization across many generations. In contrast, synthetic neopolyploids provide an opportunity to study the immediate consequences of WGD on plant morphology and physiology (Maherali *et al.*, 2009; Madlung, 2013; Spoelhof *et al.*, 2017; Van Drunen and Husband, 2018). Artificial induction of autopolyploids using antimitotic chemicals (e.g. colchicine) has been successfully carried out in both plant breeding and research (Stupar *et al.*, 2007; Vyas *et al.*, 2007; Liu *et al.*, 2011) and has provided valuable insights into the mechanisms by which polyploidization *per se* affects plant phenotypes. Discerning immediate WGD versus post-WGD phenotypic effects is possible by comparing the synthetic polyploids with parental diploids and natural polyploids (Martin and Husband, 2012; Husband *et al.*, 2016; Pavlíková *et al.*, 2017; Van Drunen and Husband, 2018). On the one hand, differences between diploids and synthetic neopolyploids in functional traits will reveal the instantaneous effects of WGD. On the other hand, comparisons between synthetic neopolyploids and naturally established polyploids might indicate which polyploidy-mediated shifts are adaptive (because they are retained in natural polyploids) and which ones are not advantageous in the long term.

*Dianthus broteri* (Caryophyllaceae) provides an excellent system for discerning the immediate effects of WGD from the subsequent evolutionary processes. This Iberian taxon encompasses the largest known autopolyploid complex within the genus (Balao *et al.*, 2009), including diploid (2*n* = 2*x* = 30), tetraploid (2*n* = 4*x* = 60), hexaploid (2*n* = 6*x* = 90), and dodecaploid (2*n* = 12*x* = 180) monocytotypic populations. This complex seems to have evolved recently (0.9–2.1 Mya) following a rapid cytotypic adaptive divergence in concert with an extensive geographical disjunction and an ecological niche differentiation within an aridity gradient, with the higher cytotypes (6*x* and 12*x*) inhabiting more restricted and extreme habitats (Balao *et al.*, 2010; López-Jurado *et al.*, 2019). Diploids occupy the most benign Mediterranean subclimate, whilst tetraploids have enhanced ecological tolerances, encompassing the most diverse environmental conditions. This niche divergence is accompanied by numerous phenotypic differences (vegetative and reproductive) between the cytotypes (Balao *et al.*, 2011), as well as different strategies regarding light-harvesting and photoprotection under temperature stress, therefore suggesting divergent adaptations (López-Jurado *et al.*, 2020). All these phenotypic differences seem to be due to a mixture of both immediate and post-WGD effects. For example, stomatal sizes vary with ploidy according to the nucleotypic effect, and some floral parts show within-cytotype differences in phenotypic optima, such as in tetraploids due to their two unrelated lineages. In addition, genome-downsizing that is linage-dependent is evident from the 1Cx values (Balao *et al.*, 2009) and higher cytotypes show increased levels of DNA methylation and epigenetic variability (Alonso *et al.*, 2016).

Within this framework, the artificial induction of *D. broteri* neopolyploids provides a potential opportunity to distinguish between the influence of WGD *per se* and subsequent adaptive changes that vary across natural populations due to reproductive isolation and divergence by selective pressures (Maherali *et al.*, 2009; Spoelhof *et al.*, 2017; Van Drunen and Husband, 2018). In this study, we established a procedure for the induction of autotetrapolyploidy by treating diploid seeds with colchicine, and we then compared several ecophysiological traits of diploids and synthetic and natural tetraploids to address the following three questions. Is genome-downsizing a direct effect of polyploidization? Are there physiological shifts directly linked to polyploidization in *D. broteri*? And to what extent can post-polyploidization mechanisms explain the functional basis of the niche differentiation observed in naturally occurring populations of *D. broteri*?

## Materials and methods

### Plant material and induction of synthetic neopolyploidy

Seeds were collected in 2018 from a diploid population (15 plants) of *Dianthus broteri* located in south-eastern Spain (37º00´N, 3º01´W). The ploidy level and DNA content of this population have previously been characterized by flow cytometry together with chromosome counts (Balao *et al.*, 2009), and its morphology and ecophysiology are known to be representative of *D. broteri* diploids (Balao *et al.*, 2011). Treatments with colchicine (Sigma-Aldrich) consisted of two different incubation times (24 h or 48 h) in combination with four different concentrations in water: 0% (w/v, control), 0.2%, 0.4%, and 0.6%. Seeds were incubated in 2-ml Eppendorf tubes in darkness on a shaker, after which they were rinsed with 25 ml of distilled water and sown on water-saturated filter paper in Petri dishes that were kept under a 12/12 h photoperiod at 25.5/17 ºC (130 µmol m^–2^ s^–1^). Germination was recorded every 2 d for 21 d, and the seedlings obtained were sown in individual 2.5-l pots filled with a mixture of a commercial organic substrate (Gramoflor) and perlite (3:1) and placed in a greenhouse with controlled temperature of 21–25 ºC, 40–60 % relative humidity (RH), and natural daylight ranging from 200–1200 μmol m^−2^ s^−1^ photosynthetic photon flux density (PPFD) incident on leaves during the day. The pots were adequately irrigated with tap water and seedling survival was checked weekly for 90 d after sowing (at which point all plants displayed sufficient vigour to be considered as definitely established). In addition, diploid and tetraploid plants from natural populations spanning the whole range of distribution of this species (Table 1) were also grown under the same greenhouse conditions. The aim of including such among-population variation was to make our results conservative (i.e. based exclusively on intrinsic characteristics of each group beyond the possible dissimilarities caused by local adaptations and genetic drift) and our conclusions robust.

**Table 1.** Population localities and numbers of plants of *D. broteri* used for the ecophysiological characterization

Cytotype	Population	Coordinates	No. of plants
2*x*	Lanjarón (Spain)	36º54´N 3º29´W	2
2*x*	Laroles (Spain)*	37º00´N 3º01´W	7
2*x*	Mecina Alfahar (Spain)	36º59´N 3º04´W	2
2*x*	Órgiva (Spain)	36º53´N 3º24´W	1
2*x*	São Brás de Alportel (Portugal)	37º09´N 7º50´W	2
4*x*_Nat_	Archidona (Spain)	37º11´N 4º31´W	2
4*x*_Nat_	La Barrosa (Spain)	36º23´N 6º07´W	3
4*x*_Nat_	Pinar de la Breña (Spain)	36º11´N 5º58´W	2
4*x*_Nat_	Doñana, Peladillo (Spain)	37º05´N 6º35´W	2
4*x*_Nat_	Puerto del Boyar (Spain)	36º45´N 5º23´W	2

All 4*x*_Neo_ (*n*=10) were induced from seeds collected in the diploid population of Laroles (marked with an asterisk).

For the ecophysiological characterization, 10 plants per group (i.e. 2*x*, 4*x*_Neo_, and 4*x*_Nat_) were randomly selected 15 d prior to analysis and placed in a controlled-environment chamber (Aralab/Fitoclima 18.000EH, Lisbon, Portugal) with a 14/10 h photoperiod (300 μmol m^−2^ s^−1^) at 25/18 °C and 40–60 % RH. This period under controlled conditions was to avoid interference by possible border effects within the greenhouse.

### Determination of DNA content and ploidy levels

The DNA content and ploidy level of the seedlings were estimated by flow cytometry (FC) following a specific protocol for *D. broteri* (Balao *et al.*, 2009). The FC measurements were conducted using a Coulter CYTOMICS FC500-MPL (Beckman Coulter, Fullerton, CA, USA) equipped with a 20 mW argon-ion laser at 488 nm. Fresh leaf material (100 mg) from glasshouse-grown *D. broteri* seedlings was used for nuclear suspensions together with *Pisum sativum* L. cv. ‘Ctirad’ as the genome size control (2C nuclear DNA = 9.09 pg). All the FC measurements showed high quality (>5000 nuclei per peak and CV <5 %) and peak means were determined through manual gating using the Kaluza Analysis 2.1 software (Beckman Coulter). DNA content (2C) was estimated for the main FC peak (phase G_0_/G_1_; Supplementary Fig. S1), which would correspond to ~1.80 pg for diploids and ~3.60 pg for tetraploids (Balao *et al.*, 2009). Mixoploidy (mixture of diploid and polyploid cells within the same tissue) was found, with some FC samples showing a second large peak representing more than 40% of the sample events, plus a third peak corresponding to nuclei in mitosis (phase G_2_) of the higher-ploidy-level cells. According to these results, each plant was classified as diploid (2*n*=2*x*), neotetraploid (2*n*=4*x*_Neo_), diploid-tetraploid mixoploid (2n=2*x*+4*x*), or tetraploid-octoploid mixoploid (2*n*=4*x*+8*x*). The mixoploids were discarded for further analysis. Monoploid genome size (1Cx) was estimated as the amount of 2C nuclear DNA divided by the ploidy level. In addition, 1Cx data for the naturally occurring tetraploids (4*x*_Nat_) were obtained from Balao *et al.* (2009).

### Stomatal measurements

Stomatal measurements were performed 80–90 d after the colchicine treatment of seeds on 11 plants for each cytotype (all the confirmed 4*x*_Neo_ plants, and randomly chosen plants for 2*x* and 4*x*_Nat_). Epidermal impressions of the region near the leaf tip were taken on the adaxial surface of the youngest fully expanded leaf using Germolene New Skin Liquid Plaster (PharmaPac, UK) and examined under a Carl-Zeiss Axiophot photomicroscope equipped with a Sony DXC-390P Exwave HAD camera. The resulting images were analysed using ImageJ v.1.51j8, and the mean stomatal density together with mean lengths and widths were determined on the basis of 10 images per plant, at 10× magnification for density and 40× for the dimensions (Balao *et al.*, 2011).

### Measurement of leaf gas exchange

A LI-COR LI-6400 infrared gas analyser in an open system equipped with a Li-6400-02B LED light source was used for instantaneous gas-exchange measurements and for constructing *A*/*C*_i_ curves. Five fully developed leaves were randomly selected for measurement on each cytotype. Net photosynthetic rate (*A*_N_), stomatal conductance (*g*_s_), and intercellular CO_2_ concentration (*C*_i_) were recorded at a PPFD of 1000 µmol m^−2^ s^−1^ (with 15% blue light to maximize stomatal aperture), vapour pressure deficit of 2.0–3.0 kPa, ambient CO_2_ concentration (*C*_a_) of 400 µmol CO_2_ mol^−1^, leaf temperature of 25±2 °C, and 50±5% RH. In addition, leaves were dark-adapted for 10 min for measurement of dark respiration (*R*_d_).

We estimated mesophyll conductance (*g*_m_) and maximum carboxylation activity of Rubisco (*V*_c,max_) by the curve-fitting method (Ethier and Livingston, 2004) and using the software package developed by Sharkey *et al.* (2007). *A*/*C*_i_ responses were measured once the leaves had reached a steady-state under the conditions described above (~20 min), at which point *C*_a_ was decreased in the following steps: 400, 350, 300, 250, 200, 150, 100, and 50 µmol mol^−1^. The chamber conditions were then restored to their initial levels and *C*_a_ was increased in the following steps to complete the curve: 500, 750, 1000, 1250, 1500, 1750, and 2000 µmol mol^−1^. At each step, gas exchange was allowed to equilibrate to avoid significant variations in Rubisco activity (generally <180 s; Long and Bernacchi, 2003). CO_2_ leakages into and out of the leaf chamber were determined using photosynthetically inactive leaves and the correction was applied to all the curves, as described previously by Flexas *et al.* (2013).

### Analysis of leaf chlorophyll fluorescence

Modulated Chlorophyll *a* fluorescence was measured in dark-adapted leaves of each cytotype using a FluorPen FP100 PAM (Photon Systems Instruments, Czech Republic). The maximum quantum efficiency of PSII (*F*_v_/*F*_m_) was determined by using a 0.8 s saturating light beam with an intensity of 8000 μmol m^−1^ s^−1^ (Schreiber *et al.*, 1986), with 10 replicates per cytotype. In addition, Kautsky curves (which indicate the fast kinetics of Chlorophyll *a*) were obtained via OJIP tests implemented in the pre-programmed protocols of the FluorPen FP100 (*n*=10). *F*_v_/*F*_m_, absorbed energy flux (ABS/CS), trapped energy flux (TR/CS), electron transport energy flux (ET/CS), and dissipated energy flux (DI/CS) per leaf cross-section, together with performance index (PI) derived from OJIP were calculated according to Strasser *et al.* (2004, 2010). These energy transduction fluxes on a leaf cross-section basis were employed due to differences in the density of reaction centres (RC/CS) among the cytotypes, according to López-Jurado *et al.* (2020). Maximum electron transport rate (ETR_max_) was also obtained using the pre-programmed rapid light curve (RLC) protocol (*n*=6), which consisted of the exposure of dark-adapted leaves (30 min) to increasing light levels (100, 200, 300, 500, and 1000 µmol m^−2^ s^−1^) and recording the quantum yield at each step.

### Statistical analyses

Data analyses were carried out using R v.3.6.3 (http://www.R-project.org/). The effects of the colchicine treatments (concentration and duration as main factors) on seed germination and seedling survival (dependent variables) were tested by fitting generalized linear models (GLMs) using a binomial error structure and the ‘logit’ link function for each dependent variable. The effectiveness of the colchicine treatments in producing viable polyploids was examined using the Fisher’s exact test. One-way ANOVA and *post hoc* Tukey’s tests were used for detecting differences between the cytotypes in the 1Cx DNA contents and the ecophysiological parameters derived from the OJIP tests and RLC protocol.

## Results

### Effects of colchicine on germination, seedling survival, and neopolyploidy induction rate

**Fig. 1.**
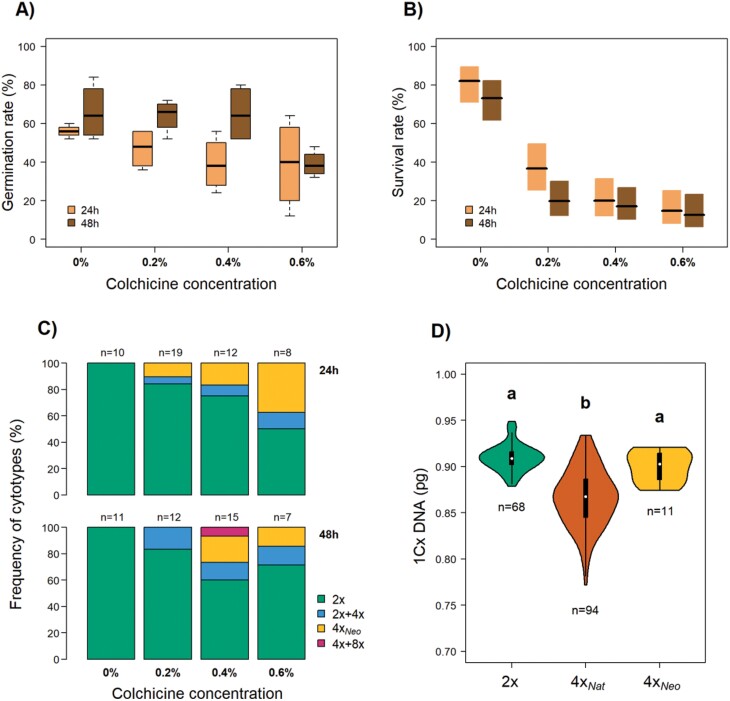
Effects of colchicine treatments on seeds of *Dianthus broteri*. Seeds were treated with colchicine at concentrations of 0, 0.2, 0.4, or 0.6% for either 24 h or 48 h. (A) Germination rate (*n*=100) and (B) survival rate of seedlings. The boxplots show the median, the interquartile range, and the non-outlier range. (C) Frequency of cytotypes obtained under the different incubation times and concentrations. (D) Monoploid genome sizes of the 2*x* diploid, the 4*x*_Nat_ natural tetraploid, and the 4*x*_Neo_ neotetraploid. In the violin plots, white circles represent the median, thick black bars correspond to the interquartile range, and the thin lines show the 95% confidence interval. Different letters indicate significant differences among the cytotypes as determined using ANOVA followed by Tukey’s test (*P*<0.05).

Germination was significantly affected by colchicine concentration and incubation time, and there was no interaction between them (Table 2). Higher concentrations of colchicine resulted in lower germination success, from 61% in the control to 39% in seeds treated with 0.6 % colchicine. Seeds incubated for 48 h showed 29.3% higher germination overall than those incubated for 24 h (Fig. 1A). Seedling survival was also significantly affected by the colchicine concentration and by the incubation time, and again there was no interaction. An increase in colchicine concentration resulted in a decrease in survival rate, from 77.5% in the control to 13.74% in seeds treated with 0.6 % colchicine; however, in contrast to germination, survival was 20.8% lower overall in seedlings from the 48 h treatment compared to 24 h (Fig. 1B).

**Table 2.** Summary of generalized linear model (GLM) results for seed germination and seedling survival in response to colchicine concentration and incubation time

Response variable	Factor	L-R χ ^2^	d.f.	*P*-value
Seed germination	Colchicine concentration	21.3072	3	<0.001
	Incubation time	14.2144	1	<0.001
	Colchicine concentration × Incubation time	7.1943	3	>0.05
Seedling survival	Colchicine concentration	82.779	3	<0.001
	Incubation time	3.935	1	<0.05
	Colchicine concentration × Incubation time	1.746	3	>0.05

L-R χ ^2^, likelihood-ratio chi-square; d.f., degrees of freedom.

A total of 94 plants were subjected to flow cytometry analysis (21 controls and 73 that survived the colchicine treatments) of which 74 were diploids (the 21 controls and 53 treated plants), 11 were tetraploids, and nine were mixoploids (eight 2*x*+4*x* and one 4*x*+8*x*). The frequency of polyploidy induction was significantly higher as the dose (i.e. concentration and incubation time) of colchicine increased (Fisher’s exact test, *P*<0.05; Fig. 1C). The control treatment invariably failed to induce polyploidization. The highest induction of neopolyploidy (tetraploids 37.5% of the established plants) was obtained at 0.6 % colchicine concentration and 24 h incubation. The highest frequency of mixoploids (17.6 % of the established plants) was obtained after 48 h.

### Genome size variation

The nuclear DNA amount of the synthetic 4*x*_Neo_ plants (3.60±0.07 pg; mean±s.d.) was about twice that of the diploids (1.82±0.03 pg), whilst the natural tetraploids showed a mean genome size of 3.47±0.13 pg. Accordingly, the monoploid DNA amount (1Cx) was significantly dependent on cytotype (*F*_2,170_=59.49, *P*<0.001; Fig. 1D). Whilst no significant differences were found when 2*x* (0.910±0.014 pg) and 4*x*_Neo_ (0.900±0.017 pg) were compared (*P*>0.05), 4*x*_Nat_ showed a significantly lower 1Cx DNA value than either of them (*P<*0.05), varying from 0.77–0.94 pg.

### Polyploidy-mediated differences in functional traits

**Fig. 2.**
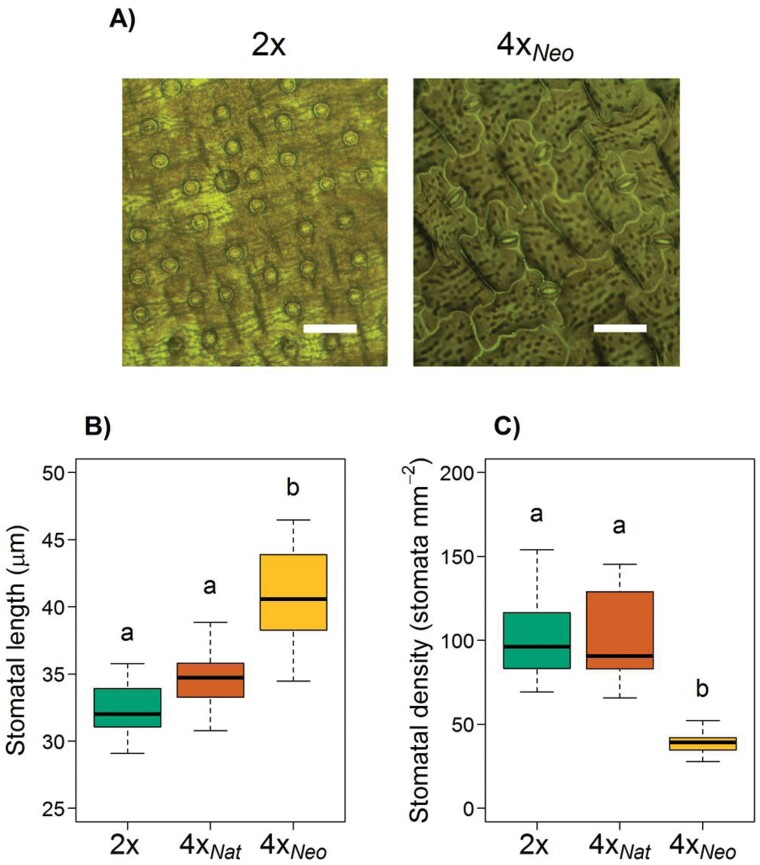
Stomatal sizes of diploids (2*x*), natural tetraploids (4*x*_Nat_), and colchicine-induced neotetraploids (4*x*_Neo_) of *Dianthus broteri*. (A) Epidermal impressions of leaves of 2*x* and 4*x*_Neo_ plants (4*x*_Nat_ was similar 2*x* and is therefore not shown). Scale bars are 100 µm. (B) Stomatal length and (C) density of the three cytotypes. The boxplots show the median, the interquartile range, and the non-outlier range. Different letters indicate significant differences among cytotypes as determined using ANOVA followed by Tukey’s test (*P*<0.05).

Stomatal traits were affected differently by polyploidization (Fig. 2). Comparisons among the cytotypes revealed significant differences in stomatal length (*F*_2,30_=25.79, *P*<0.001) and width (*F*_2,30_=9.61, *P*<0.001), as well as density (*F*_2,30_=28.49, *P*<0.001). Whilst length, width, and density were indistinguishable between 2*x* and 4*x*_Nat_ (*P*>0.05), varying between 29–39 μm, 19–28 μm, and 66–154 mm^−2^, respectively, the synthetic neotetraploids showed significantly longer (40.58±3.77 μm; *P<*0.05) and wider (28.24±2.97 μm; *P<*0.05) stomata than either of them, but had a lower density (38.91±6.91 mm^−2^; *P<*0.05). Notably, the synthetic tetraploids showed an increased variation in stomatal length (CV 6.1%, 6.4%, and 9.3% in 2*x*, 4*x*_Nat_, and 4*x*_Neo_, respectively), but a more consistent density (CV 26.3%, 27.4%, and 17.8% in 2*x*, 4*x*_Nat_, and 4*x*_Neo_, respectively).

**Fig. 3.**
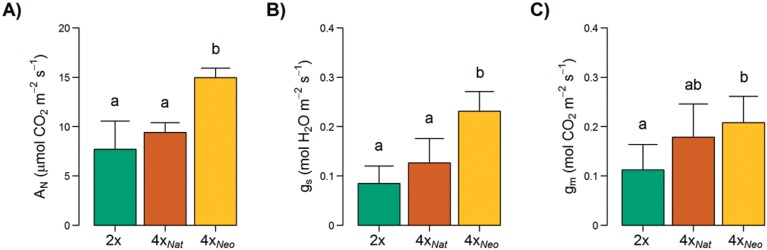
Effects of polyploidization in *Dianthus broteri* on leaf gas exchange. Measurements were taken on diploids (2*x*), natural tetraploids (4*x*_Nat_), and colchicine-induced neotetraploids (4*x*_Neo_). (A) Net photosynthetic rate (*A*_N_), (B) stomatal conductance (*g*_s_), and (C) and mesophyll conductance (*g*_m_). Data are means (±SD), *n*=5. Different letters indicate significant differences among cytotypes as determined using ANOVA followed by Tukey’s test (*P*<0.05).

Photosynthetic performance was also affected by polyploidization, but 4*x*_Nat_ did not always display the same trends than 4*x*_Neo_. Analysis of leaf gas exchange showed that the synthetic neopolyploids had a significant increase in net photosynthetic rate (*A*_N_; *F*_2,13_=14.72, *P*<0.001), stomatal conductance (*g*_s_; *F*_2,13_=17.71, *P*<0.001), and mesophyll conductance (*g*_m_; *F*_2,11_=4.48, *P*<0.05) in comparison to the diploids and natural tetraploids, which showed similar values to each other (Fig. 3). *A*_N_ and *g*_s_ were almost doubled in 4*x*_Neo_ compared to 2*x* and 4*x*_Nat_ (*P*<0.05), which had similar values. The 4*x*_Neo_ plants also showed ~2-fold greater *g*_m_ than 2*x* (*P*<0.05), whilst 4*x*_Nat_ showed intermediate values between those of 2*x* and 4*x*_Neo_. Although the results showed broadly similar patterns, no significant differences were found among the cytotypes for the other photosynthetic parameters that were measured, namely intercellular CO_2_ concentration (*C*_i_), maximum rate of Rubisco activity (*V*_c,max_), and the rate of dark respiration (*R*_d_) (Supplementary Fig. S2; ANOVA, *P*>0.05).

**Fig. 4.**
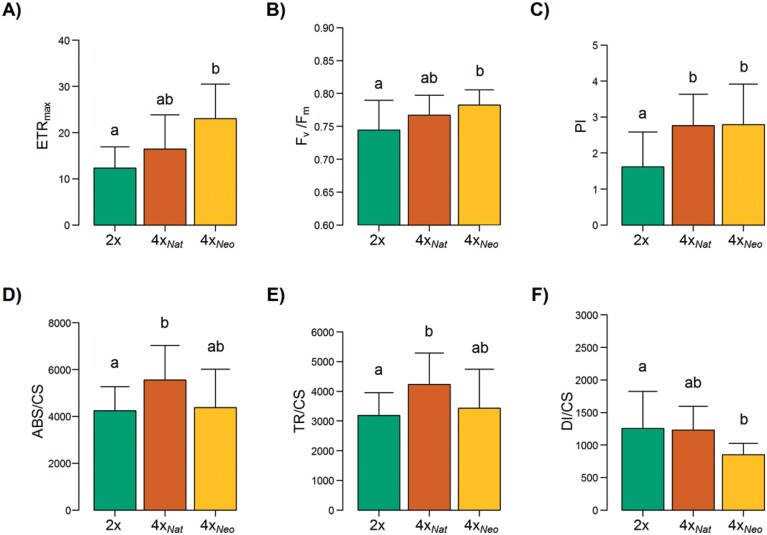
Effects of polyploidization in *Dianthus broteri* on leaf chlorophyll fluorescence. Measurements were taken on diploids (2*x*), natural tetraploids (4*x*_Nat_), and colchicine-induced neotetraploids (4*x*_Neo_). (A) Maximum electron transport rate (ETR_max_), (B) maximum quantum efficiency of PSII (*F*_v_/*F*_m_), (C) performance index (PI), (D) absorbed energy flux (ABS/CS), (E) trapped energy flux (TR/CS), and (F) dissipated energy flux (DI/CS) per leaf cross-section. Data are means (±SD), *n*=10, except (A) where *n*=6. Different letters indicate significant differences among cytotypes as determined using ANOVA followed by Tukey’s test (*P*<0.05).

Analysis of leaf chlorophyll fluorescence also showed several distinct patterns of variation between the cytotypes (Fig. 4). Significant differences were found for maximum electron transport rate (ETR_max_; *F*_2,20_=5.64, *P*<0.05) and maximum quantum efficiency of PSII (*F*_v_/*F*_m_; *F*_2,48_=4.17, *P*<0.05). In both cases, higher values were found in 4*x*_Neo_ compared to 2*x*, whilst vales in 4*x*_Nat_ were intermediate (Fig. 4A, B), indicating a reduction in the polyploidy-mediated direct effect on these traits. Such an after-WGD reduction was not found in the performance index (PI), with 4*x*_Neo_ and 4*x*_Nat_ showing higher values (71.5% on average) than 2*x* (*F*_2,47_=8.70, *P*<0.001; Fig. 4C).

A different pattern was observed for the energy transduction fluxes. We did not observe a direct effect of WGD but a post-polyploidization divergence was found for the absorbed energy flux per cross-section (ABS/CS; *F*_2,47_=4.87, *P*<0.05; Fig. 4D). The values for 2*x* and 4*x*_Neo_ were similar (*P*>0.05), whilst 4*x*_Nat_ showed a 30.8% increase in ABS/CS compared to 2*x* (*P*<0.01). No significant difference was found between 4*x*_Nat_ and 4*x*_Neo_ (*P*>0.05). The same post-WGD divergence was also observed for the trapped energy flux per cross-section (TR/CS; *F*_2,47_=5.31, *P*<0.01; Fig. 4E), with 4*x*_Nat_ showing a 33.0% increase compared to the diploids. A similar trend was found in the electron transport energy flux per cross-section (ET/CS), but there were no significant differences among the cytotypes (*F*_2,47_=1.75, *P*>0.05), with mean values of 1717, 1960, and 2065 for 2*x*, 4*x*_Neo_, and 4*x*_Nat_, respectively (Supplementary Fig. S2). Finally, the dissipated energy flux per cross-section (DI/CS) was only marginally affected by chromosome doubling (*F*_2,45_=2.57, *P*=0.09),with 4*x*_Neo_ showing ~0.3-fold lower dissipation values than 2*x* and 4*x*_Nat_ (Fig. 4F).

## Discussion

Exposure to colchicine through seed imbibition was an efficient method for obtaining synthetic neopolyploids of *Dianthus broteri*, and our results could have implications for the optimization of autopolyploidy induction in general (Fig. 1). Despite polyploidization increasing in parallel with colchicine concentration, large reductions in germination and seedling survival at high dosages made treatments with intermediate doses more suitable for inducing neopolyploidy. This was consistent with results of previous studies (Thiebaut and Kasha, 1978; Chen *et al.*, 2006; Lehrer *et al.*, 2008; Omidbaigi *et al.*, 2010; Xing *et al.*, 2011; Pavlíková *et al.*, 2017; Sadat Noori *et al.*, 2017), although the different methodologies (different antimitotic agents and/or different target tissues such as shoot apical meristem or callus) and the species-specific responses to colchicine do not make it possible to establish a general protocol for neopolyploidy induction.

Quantification of nuclear DNA through flow cytometry provides a practical method for quickly determining ploidy levels (Balao *et al.*, 2009) and it was also effective for discerning mixoploidy (Fig. 1C). Analysis of the monoploid genome size (1Cx) revealed remarkable genome size stability in nascent neopolyploids but a moderate genomic diploidization in the natural tetraploid populations (Fig. 1D), as previously shown by Balao *et al.* (2009). This is a common phenomenon in mesopolyploids (Dodsworth *et al.*, 2016; Mandákova *et al*., 2010) and could be mainly due to gene loss and chromosomal rearrangements contributing to restore successful cyto–nuclear interactions (Sharbrough *et al.*, 2017). Although genetic change seems to be subtle (but spread locally) in this *D. broteri* polyploid complex due to rapid diversification (Balao *et al.*, 2010), genomic adjustments could have played a crucial role in adaptation after WGD, not only providing genomic stability but also accounting for transcriptomic distinctness, which frequently results in phenotypic novelty and ecophysiological differentiation (Chen *et al.*, 2007; Chelaifa *et al.*, 2010; Soltis *et al.*, 2014; Dodsworth *et al.*, 2016). In this regard, genomic diploidization could have boosted local adaptations following the post-WGD niche expansion, and fostered the remarkable ecological features of *D. broteri* tetraploids, which show greater niche breadth compared with the rest of the cytotypes (López-Jurado *et al.*, 2019) and also distinct light-harvesting and photoprotection strategies (López-Jurado *et al.*, 2020).

The few previous studies that have examined the effects of autopolyploidy on plant physiology have focused on synthetic neopolyploids (Stupar *et al.*, 2007; Liu *et al.*, 2011; Dong *et al.*, 2017; Wei *et al.*, 2020) or on divergence in natural populations (Balao *et al.*, 2011; Manzaneda *et al.*, 2012; Thompson *et al.*, 2014; Pacey *et al.*, 2020). In contrast, our approach was able to distinguish not just the post-WGD effects on several physiological and development traits, but also the immediate effects that help to explain the divergent evolution of the ecological niche in the polyploids. For example, it is well established that stomatal characteristics have a great impact on the physiology of land plants, and climatic conditions usually operate as selective forces for these traits (Veselý *et al.*, 2020). Our data indicated that autopolyploidization in *D. broteri* directly modified the size of guard cells (i.e. the ‘gigas effect’), resulting in larger stomata with greater aperture area available for gas exchange (Fig. 2). Conversely, the density of stomata immediately after WGD decreased, but interestingly tetraploid populations in the wild did not show these changes in stomatal size and density. A reduction in stomatal size and an increase in density after WGD has also been shown in other polyploid systems (e.g. Maherali *et al.*, 2009; Münzbergová, 2017). Thus, a ‘phenotypic diploidization’ could occur after WGD on these stomatal traits, which become diploid-like either due to directional selection after establishment (Vyas *et al.*, 2007) or because the establishment of polyploid individuals with unfit stomatal characteristics is prevented (Comai, 2005; Soltis *et al.*, 2014). In addition, stomatal downsizing in 4*x*_Nat_*D. broteri*, together with an increased number of stomata per unit area, would be related to the genome downsizing (i.e. genomic diploidization) in the natural tetraploids. Variation in cell size (related to the loss of DNA content) will play a crucial role in optimizing gas exchange, potential carbon gain, and water use after WGD (Simonin and Roddy, 2018; Lawson and Vialet-Chabrand, 2019; Roddy *et al.*, 2020). Given the fact that higher stomatal densities have been associated with more arid climates (Carlson *et al.*, 2016), the phenotypic and genomic diploidization in the higher-order neopolyploids (6*x*, 12*x*) of *D. broteri* could have also triggered adaptation to harsh environments (Balao *et al.*, 2011). In addition, the remarkable within-cytotype variation of stomatal size in naturally occurring 2*x* and 4*x* populations (Fig. 2) suggests that local adaptations in *D. broteri* might reside in adjusting stomatal density rather than stomatal size (Wei *et al.*, 2019).

Physiological traits also showed a mixture of immediate and post-WGD effects. However, we did not find any differences between cytotypes (either synthetic or natural polyploids) in maximum carboxylation rate of Rubisco (*V*_c,max_), dark respiration (*R*_d_), CO_2_ concentration in the substomatal cavities (*C*_i_), or the electron transport energy flux per leaf cross-section (ET/CS) (Supplementary Fig. S2), so the few (if any) changes following WGD would be deleterious (e.g. under purifying selection). Interestingly, a previous study showed significant down-regulation in linear electron transport (i.e. ET/CS) only in the higher-ploidy levels (6*x* and 12*x*) of the *D. broteri* complex, suggesting a photoprotective adaptation to their stressful ecological niches (López-Jurado *et al.*, 2020).

Phenotypic diploidization was evident in the stomatal and mesophyll conductances (*g*_s_ and *g*_m_; Fig. 3) and hence the increases gained through WGD were not maintained in 4*x*_Nat_. In agreement with our results (i.e. 4*x*_Neo_ having higher conductances than 2*x*), neopolyploids frequently display increased *g*_s_ and *g*_m._ (Vyas *et al.*, 2007; Wang *et al.*, 2019; Monda *et al.*, 2016). This might be partially explained by structural differences between the ploidy levels, with greater stomatal area and larger intercellular spaces in the neopolyploids. Nevertheless, other non-structural factors directly affected by WGD could help to explain the differences in conductances, such as osmotic adjustment and metabolic regulation of stomatal opening (Mouhaya *et al.*, 2010; Monda *et al.*, 2016). It is worth noting that our physiological measurements were taken under optimal conditions and that a different picture might be seen under stressful ones (López-Jurado *et al.*, 2020). The tendency of a return to diploid-like *g*_s_ and *g*_m_ values after WGD has not been previously reported to our knowledge, and a single study comparing diploids, neopolyploids, and established polyploids found that increases in *g*_s_ and *g*_m_ observed immediately after WGD tended to then increase even further (Vyas *et al.*, 2007). However, this diploidization of conductances could accompany the structural diploidization of the stomata. Higher densities of smaller stomata are advantageous for gas exchange because they result in shorter diffusion path lengths (Franks and Beerling, 2009), and they can protect plants from xylem embolisms as well as facilitate the reduction of water loss (Li *et al.*, 1996; te Beest *et al.*, 2012).

The net photosynthetic rate (*A*_N_), which correlates with *g*_s_ and *g*_m_ (among other parameters), showed higher values for 4*x*_Neo_ whilst it did not differ between 2*x* and 4*x*_Nat_ (Fig. 3). A greater photosynthetic capacity in combination with faster growth rate is common in neopolyploids (Ramsey and Schemske, 2002; Saleh *et al.*, 2008; Münzbergová, 2017) and seem to be the rule in established mesopolyploids (Hull-Sanders *et al.*, 2009; Ježilová *et al.*, 2015; Liao *et al.*, 2016). Accordingly, the noticeably lower net photosynthetic rate that we observed in natural tetraploids (similar to that in the diploids) would also be related to the genomic diploidization and the consequent stomatal adaptations to optimize the balance between CO_2_ acquisition and water losses under a wide range of environmental conditions (Simonin and Roddy, 2018; Roddy *et al.*, 2020).

The influence of phenotypic diploidization was also observed for the efficiency of the PSII apparatus, as indicated by the differences between the cytotypes in several chlorophyll fluorescence parameters (Fig. 4) (Maxwell and Johnson, 2000). The 4*x*_Neo_ plants showed higher values for maximum electron transport rate (ETR_max_) and maximum quantum efficiency of PSII (*F*_v_/*F*_m_), and we also found that WGD *per se* decreased the dissipated energy flux per leaf cross-section (DI/CS). These results indicated that much of the energy absorbed by the 4*x*_Neo_ plants would have been transformed in their photosystems and directed to the photochemical pathway (Flexas *et al.*, 2013), which is consistent with their greater CO_2_ assimilation capacity, as discussed above. Conversely, the higher DI/CS value in the natural populations could reflect a protective mechanism against the high irradiance typical of the Mediterranean dry season found at the localities inhabited by *D. broteri* (López-Jurado *et al.*, 2019, 2020). Thus, environmental constraints may favour selection towards a return to the original diploid DI/CS values (i.e. phenotypic diploidization).

Notwithstanding this pattern of phenotypic diploidization being observed in leaf gas-exchange and in some leaf chlorophyll fluorescence parameters, other photosynthetically relevant variables showed an increase in post-WGD differentiation. While López-Jurado *et al.* (2020) did not detect significant differences between 2*x* and 4*x* cytotypes at non-stressful temperatures for absorbed and trapped energy fluxes per leaf cross-section (ABS/CS and TR/CS, respectively), our design allowed us to distinguish them (Fig. 4). Although there was not a clear immediate effect of WGD on these parameters, a post-WGD selection for elevated energy fluxes was revealed. A possible mechanistic explanation for the increases in ABS/CS and TR/CS in the natural tetraploids would be a preferential retention of dosage-sensitive genes encoding components of both PSI and PSII, which would cause increased gene expression during the diploidization process (Coate *et al.*, 2011). Synthetic tetraploids would display similar values to their progenitors because the WGD would maintain metabolic homeostasis (i.e. not alter molecular stoichiometry; He *et al.*, 2013). An alternative explanation for the enhanced ABS/CS and TR/CS in 4*x*_Nat_ would be the neofunctionalization of duplicate genes encoding components associated with the two photosystems, with the alterations potentially enhancing their functionality (Coate and Doyle, 2013). It was notable that despite these differences in ABS/CS, TR/CS, and DI/CS, the tetraploids conserved the photochemical superiority conferred by autopolyploidization *per se* (as mirrored by the values of performance index; Fig. 4C), indicating the necessity for natural polyploids of maintaining this feature.

Our study has highlighted the relative importance of WGD *per se* and post-WGD selection in determining the distinct phenotypes of the 2*x* and 4*x* cytotypes of *D. broteri.* The polyploidy-related shifts in ecophysiological traits that we have identified could provide selective advantages that lead to the initial short-term survival and establishment of polyploids, which usually occupy new niches out of the range of the diploid (te Beest *et al.*, 2012). Although evidence for several alternative niche-evolution patterns has been found (Glennon *et al.*, 2014; Pegoraro *et al.*, 2019), divergence is one of the most commonly described in polyploid complexes (Thompson *et al.*, 2014; López-Jurado *et al.*, 2019). In *D. broteri*, the increased photosynthetic capacity observed in the synthetic neopolyploids (in comparison to diploids) would have the potential to facilitate their establishment through the exploitation of new niches, and would thus explain the wider ecological range of the tetraploids (López-Jurado *et al.*, 2019).

In summary, the results of our study not only suggest that many important plant functional traits are directly affected by polyploidization, but they also reveal that the process of phenotypic diploidization can lead to the creative role of polyploidization being underestimated. Thus, phenotypic novelty in neopolyploids might be constrained by regulatory mechanisms related to physiology as well as by other evolutionary processes related to environmental pressures.

## Supplementary data

The following supplementary data are available at *JXB* online.

Fig. S1. Flow cytometry analyses of the plants arising from colchicine-treated seeds.

Fig. S2. Intercellular CO_2_ concentration, maximum carboxylation rate of Rubisco, dark respiration, and electron transport energy flux per leaf cross-section in the different cytotypes..

erab179_suppl_Supplementary_Figures_S1-S2

## Data Availability

The data supporting the findings of this study are available from the corresponding author, J.J. Domínguez-Delgado, upon request.

## Abbreviations

ABS/CS: absorbed energy flux per leaf cross-section
*A*_N_: net photosynthetic rate
*C*_a_: ambient CO_2_ concentration
*C*_i_: intercellular CO_2_ concentration
DI/CS: dissipated energy flux per leaf cross-section
ET/CS: electron transport energy flux per leaf cross-section
ETR_max_: maximum electron transport rate
FC: flow cytometry
*F*_v_/*F*_m_: maximum quantum efficiency of PSII
g_m_: mesophyll conductance
*g*_*s*_: stomatal conductance
PI: performance index
PPFD: photosynthetic photon flux density
RC/CS: reaction centres per cross-section
*R*_d_: dark respiration
TR/CS: trapped energy flux per leaf cross-section
*V*_c,max_: maximum carboxylation rate of Rubisco
WGD: whole-genome duplication

## References

[CIT0001] Alonso C, Balao F, Bazaga P, Pérez R. 2016. Epigenetic contribution to successful polyploidizations: variation in global cytosine methylation along an extensive ploidy series in *Dianthus broteri* (Caryophyllaceae). New Phytologist 212, 571–576. 10.1111/nph.14138 27483440

[CIT0002] Balao F, Casimiro-Soriguer R, Talavera M, Herrera J, Talavera S. 2009. Distribution and diversity of cytotypes in *Dianthus broteri* as evidenced by genome size variations. Annals of Botany 104, 965–973. 19633312 10.1093/aob/mcp182 PMC2749526

[CIT0003] Balao F, Herrera J, Talavera S. 2011. Phenotypic consequences of polyploidy and genome size at the microevolutionary scale: a multivariate morphological approach. New Phytologist 192, 256–265. 10.1111/j.1469-8137.2011.03787.x 21651562

[CIT0004] Balao F, Valente LM, Vargas P, Herrera J, Talavera S. 2010. Radiative evolution of polyploid races of the Iberian carnation *Dianthus broteri* (Caryophyllaceae). New Phytologist 187, 542–551. 10.1111/j.1469-8137.2010.03280.x 20456054

[CIT0005] Carlson JE, Adams CA, Holsinger KE. 2016. Intraspecific variation in stomatal traits, leaf traits and physiology reflects adaptation along aridity gradients in a South African shrub. Annals of Botany 117, 195–207. 26424782 10.1093/aob/mcv146 PMC4701147

[CIT0006] Chelaifa H, Monnier A, Ainouche M. 2010. Transcriptomic changes following recent natural hybridization and allopolyploidy in the salt marsh species *Spartina* × *townsendii* and *Spartina anglica* (Poaceae). New Phytologist 186, 161–174. 10.1111/j.1469-8137.2010.03179.x 20149114

[CIT0007] Chen LP, Wang YJ, Zhao M. 2006. *In vitro* induction and characterization of tetraploid *Lychnis senno* Siebold et Zucc. HortScience 41, 759–761.

[CIT0008] Chen ZJ . 2007. Genetic and epigenetic mechanisms for gene expression and phenotypic variation in plant polyploids. Annual Review of Plant Biology 58, 377–406. 10.1146/annurev.arplant.58.032806.103835 PMC1949485 17280525

[CIT0009] Chen ZJ, Ha M, Soltis D. 2007. Polyploidy: genome obesity and its consequences. New Phytologist 174, 717–720. 10.1111/j.1469-8137.2007.02084.x PMC1950720 17504455

[CIT0010] Cheng F, Wu J, Cai X, Liang J, Freeling M, Wang X. 2018. Gene retention, fractionation and subgenome differences in polyploid plants. Nature Plants 4, 258–268. 29725103 10.1038/s41477-018-0136-7

[CIT0011] Coate JE, Doyle JJ. 2013. Genomics and transcriptomics of photosynthesis in polyploids. In: Chen ZJ, Birchler JA, eds. Polyploid and hybrid genomics. Hoboken, NJ: Wiley, 153–169.

[CIT0012] Coate JE, Schlueter JA, Whaley AM, Doyle JJ. 2011. Comparative evolution of photosynthetic genes in response to polyploid and nonpolyploid duplication. Plant Physiology 155, 2081–2095. 21289102 10.1104/pp.110.169599 PMC3091097

[CIT0013] Comai L . 2005. The advantages and disadvantages of being polyploid. Nature Reviews Genetics 6, 836–846. 10.1038/nrg1711 16304599

[CIT0014] Dodsworth S, Chase MW, Leitch AR. 2016. Is post-polyploidization diploidization the key to the evolutionary success of angiosperms? Botanical Journal of the Linnean Society 180, 1–5.

[CIT0015] Dong B, Wang H, Liu T, Cheng P, Chen Y, Chen S, Guan Z, Fang W, Jiang J, Chen F. 2017. Whole genome duplication enhances the photosynthetic capacity of *Chrysanthemum nankingense*. Molecular Genetics and Genomics 292, 1247–1256. 28674743 10.1007/s00438-017-1344-y

[CIT0016] Doyle JJ, Coate JE. 2019. Polyploidy, the nucleotype, and novelty: the impact of genome doubling on the biology of the cell. International Journal of Plant Sciences 180, 1–52.

[CIT0017] Ethier GJ, Livingston NJ. 2004. On the need to incorporate sensitivity to CO_2_ transfer conductance into the Farquhar–von Caemmerer–Berry leaf photosynthesis model. Plant, Cell & Environment 27, 137–153.

[CIT0018] Flexas J, Niinemets U, Gallé A, et al. 2013. Diffusional conductances to CO_2_ as a target for increasing photosynthesis and photosynthetic water-use efficiency. Photosynthesis Research 117, 45–59. 23670217 10.1007/s11120-013-9844-z

[CIT0019] Franks PJ, Beerling DJ. 2009. Maximum leaf conductance driven by CO_2_ effects on stomatal size and density over geologic time. Proceedings of the National Academy of Sciences, USA 106, 10343–10347. 10.1073/pnas.0904209106 PMC2693183 19506250

[CIT0020] Glennon KL, Ritchie ME, Segraves KA. 2014. Evidence for shared broad-scale climatic niches of diploid and polyploid plants. Ecology Letters 17, 574–582. 24818236 10.1111/ele.12259

[CIT0021] Glick L, Sabath N, Ashman TL, Goldberg E, Mayrose I. 2016. Polyploidy and sexual system in angiosperms: is there an association? American Journal of Botany 103, 1223–1235. 27352832 10.3732/ajb.1500424

[CIT0022] He Y, Dai S, Dufresne CP, Zhu N, Pang Q, Chen S. 2013. Integrated proteomics and metabolomics of Arabidopsis acclimation to gene-dosage dependent perturbation of isopropylmalate dehydrogenases. PLoS ONE 8, e57118. 23533573 10.1371/journal.pone.0057118 PMC3606340

[CIT0023] Hull-Sanders HM, Johnson RH, Owen HA, Meyer GA. 2009. Effects of polyploidy on secondary chemistry, physiology, and performance of native and invasive genotypes of *Solidago gigantea* (Asteraceae). American Journal of Botany 96, 762–770. 21628231 10.3732/ajb.0800200

[CIT0024] Husband BC, Baldwin SJ, Sabara HA. 2016. Direct vs. indirect effects of whole-genome duplication on prezygotic isolation in *Chamerion angustifolium*: implications for rapid speciation. American Journal of Botany 103, 1259–1271. 27440792 10.3732/ajb.1600097

[CIT0025] Ježilová E, Nožková-Hlaváčková V, Duchoslav M. 2015. Photosynthetic characteristics of three ploidy levels of *Allium oleraceum* L. (Amaryllidaceae) differing in ecological amplitude. Plant Species Biology 30, 212–224.

[CIT0026] Landis JB, Soltis DE, Li Z, Marx HE, Barker MS, Tank DC, Soltis PS. 2018. Impact of whole-genome duplication events on diversification rates in angiosperms. American Journal of Botany 105, 348–363. 29719043 10.1002/ajb2.1060

[CIT0027] Lawson T, Vialet-Chabrand S. 2019. Speedy stomata, photosynthesis and plant water use efficiency. New Phytologist 221, 93–98. 10.1111/nph.15330 29987878

[CIT0028] Lehrer JM, Brand MH, Lubell JD. 2008. Induction of tetraploidy in meristematically active seeds of Japanese barberry (*Berberis thunbergii* var. *atropurpurea*) through exposure to colchicine and oryzalin. Scientia Horticulturae 119, 67–71.

[CIT0029] Levin DA . 1975. Minority cytotype exclusion in local plant populations. Taxon 24, 35–43.

[CIT0030] Levin DA . 2002. The role of chromosomal change in plant evolution. Oxford: Oxford University Press.

[CIT0031] Li WL, Berlyn GP, Ashton PMS. 1996. Polyploids and their structural and physiological characteristics relative to water deficit in *Betula papyrifera* (Betulaceae). American Journal of Botany 83, 15–20.

[CIT0032] Liao T, Cheng S, Zhu X, Min Y, Kang X. 2016. Effects of triploid status on growth, photosynthesis, and leaf area in *Populus*. Trees - Structure and Function 30, 1137–1147.

[CIT0033] Liu S, Chen S, Chen Y, Guan Z, Yin D, Chen F. 2011. *In vitro* induced tetraploid of *Dendranthema nankingense* (Nakai) Tzvel. shows an improved level of abiotic stress tolerance. Scientia Horticulturae 127, 456–465.

[CIT0034] Long SP, Bernacchi CJ. 2003. Gas exchange measurements, what can they tell us about the underlying limitations to photosynthesis? Procedures and sources of error. Journal of Experimental Botany 54, 2393–2401. 14512377 10.1093/jxb/erg262

[CIT0035] López-Jurado J, Balao F, Mateos-Naranjo E. 2020. Polyploidy-mediated divergent light-harvesting and photoprotection strategies under temperature stress in a Mediterranean carnation complex. Environmental and Experimental Botany 171, 103956

[CIT0036] López-Jurado J, Mateos-Naranjo E, Balao F. 2019. Niche divergence and limits to expansion in the high polyploid *Dianthus broteri* complex. New Phytologist 222, 1076–1087. 10.1111/nph.15663 30585629

[CIT0037] Madlung A . 2013. Polyploidy and its effect on evolutionary success: old questions revisited with new tools. Heredity 110, 99–104. 23149459 10.1038/hdy.2012.79 PMC3554449

[CIT0038] Madlung A, Tyagi AP, Watson B, Jiang H, Kagochi T, Doerge RW, Martienssen R, Comai L. 2005. Genomic changes in synthetic *Arabidopsis* polyploids. The Plant Journal 41, 221–230. 15634199 10.1111/j.1365-313X.2004.02297.x

[CIT0039] Maherali H, Walden AE, Husband BC. 2009. Genome duplication and the evolution of physiological responses to water stress. New Phytologist 184, 721–731. 10.1111/j.1469-8137.2009.02997.x 19703115

[CIT0040] Mandáková T, Joly S, Krzywinski M, Mummenhoff K, Lysak MA. 2010. Fast diploidization in close mesopolyploid relatives of *Arabidopsis*. The Plant Cell 22, 2277–2290. 20639445 10.1105/tpc.110.074526 PMC2929090

[CIT0041] Manzaneda AJ, Rey PJ, Bastida JM, Weiss-Lehman C, Raskin E, Mitchell-Olds T. 2012. Environmental aridity is associated with cytotype segregation and polyploidy occurrence in *Brachypodium distachyon* (Poaceae). New Phytologist 193, 797–805. 10.1111/j.1469-8137.2011.03988.x PMC3257369 22150799

[CIT0042] Martin SL, Husband BC. 2012. Whole genome duplication affects evolvability of flowering time in an autotetraploid plant. PLoS ONE 7, e44784. 23028620 10.1371/journal.pone.0044784 PMC3447006

[CIT0043] Masterson J . 1994. Stomatal size in fossil plants: evidence for polyploidy in majority of angiosperms. Science 264, 421–424. 17836906 10.1126/science.264.5157.421

[CIT0044] Maxwell K, Johnson GN. 2000. Chlorophyll fluorescence—a practical guide. Journal of Experimental Botany 51, 659–668. 10938857 10.1093/jxb/51.345.659

[CIT0045] Monda K, Araki H, Kuhara S, et al. 2016. Enhanced stomatal conductance by a spontaneous *Arabidopsis* tetraploid, Me-0, results from increased stomatal size and greater stomatal aperture. Plant Physiology 170, 1435–1444.m 26754665 10.1104/pp.15.01450 PMC4775119

[CIT0046] Mouhaya W, Allario T, Brumos J, Andres F, Froelicher Y, Luro F, Tálon M, Ollitrault P, Morillon R. 2010. Sensitivity to high salinity in tetraploid citrus seedlings increases with water availability and correlates with expression of candidate genes. Functional Plant Biology 37, 674– 685.

[CIT0047] Münzbergová Z . 2017. Colchicine application significantly affects plant performance in the second generation of synthetic polyploids and its effects vary between populations. Annals of Botany 120, 329–339. 28633349 10.1093/aob/mcx070 PMC5737759

[CIT0048] Münzbergová Z, Haisel D. 2019. Effects of polyploidization on the contents of photosynthetic pigments are largely population-specific. Photosynthesis Research 140, 289–299. 30413987 10.1007/s11120-018-0604-y

[CIT0049] Omidbaigi R, Saba Y, Hassani ME, Sara Y. 2010. Induction of autotetraploidy in dragonhead (*Dracocephalum moldavica* L.) by colchicine treatment. Journal of Fruit and Ornamental Plant Research 18, 23–35.

[CIT0050] Pacey EK, Maherali H, Husband BC. 2020. The influence of experimentally induced polyploidy on the relationships between endopolyploidy and plant function in *Arabidopsis thaliana*. Ecology and Evolution 10, 198–216. 31988723 10.1002/ece3.5886 PMC6972801

[CIT0051] Pandit MK, Pocock MJO, Kunin WE. 2011. Ploidy influences rarity and invasiveness in plants. Journal of Ecology 99, 1108–1115.

[CIT0052] Pavlíková Z, Paštová L, Münzbergová Z. 2017. Synthetic polyploids in *Vicia cracca*: methodology, effects on plant performance and aneuploidy. Plant Systematics and Evolution 303, 827–839.

[CIT0053] Pegoraro L, De Vos JM, Cozzolino S, Scopece G. 2019. Shift in flowering time allows diploid and autotetraploid *Anacamptis pyramidalis* (Orchidaceae) to coexist by reducing competition for pollinators. Botanical Journal of the Linnean Society 191, 274–284.

[CIT0054] Ramsey J, Schemske DW. 2002. Neopolyploidy in flowering plants. Annual Review of Ecology and Systematics 33, 589–639.

[CIT0055] Rice A, Šmarda P, Novosolov M, Drori M, Glick L, Sabath N, Meiri S, Belmaker J, Mayrose I. 2019. The global biogeography of polyploid plants. Nature Ecology & Evolution 3, 265–273. 30697006 10.1038/s41559-018-0787-9

[CIT0056] Roddy AB, Théroux-Rancourt G, Abbo T, et al 2020. The scaling of genome size and cell size limits maximum rates of photosynthesis with implications for ecological strategies. International Journal of Plant Sciences 181, 75–87.

[CIT0057] Sadat Noori SA, Norouzi M, Karimzadeh G, Shirkool K, Niazian M. 2017. Effect of colchicine-induced polyploidy on morphological characteristics and essential oil composition of ajowan (*Trachyspermum ammi* L.). Plant Cell, Tissue and Organ Culture 130, 543–551.

[CIT0058] Saleh B, Allario T, Dambier D, Ollitrault P, Morillon R. 2008. Tetraploid citrus rootstocks are more tolerant to salt stress than diploid. Comptes Rendus Biologies 331, 703–710. 18722990 10.1016/j.crvi.2008.06.007

[CIT0059] Schreiber U, Schliwa U, Bilger W. 1986. Continuous recording of photochemical and non-photochemical chlorophyll fluorescence quenching with a new type of modulation fluorometer. Photosynthesis Research 10, 51–62. 24435276 10.1007/BF00024185

[CIT0060] Sharbrough J, Conover JL, Tate JA, Wendel JF, Sloan DB. 2017. Cytonuclear responses to genome doubling. American Journal of Botany 104, 1277–1280. 29885242 10.3732/ajb.1700293

[CIT0061] Sharkey TD, Bernacchi CJ, Farquhar GD, Singsaas EL. 2007. Fitting photosynthetic carbon dioxide response curves for C_3_ leaves. Plant, Cell & Environment 30, 1035–1040. 10.1111/j.1365-3040.2007.01710.x 17661745

[CIT0062] Simonin KA, Roddy AB. 2018. Genome downsizing, physiological novelty, and the global dominance of flowering plants. PLoS Biology 16, e2003706. 29324757 10.1371/journal.pbio.2003706 PMC5764239

[CIT0063] Soltis DE, Albert VA, Leebens-Mack J, Bell CD, Paterson AH, Zheng C, Sankoff D, Depamphilis CW, Wall PK, Soltis PS. 2009. Polyploidy and angiosperm diversification. American Journal of Botany 96, 336–348. 21628192 10.3732/ajb.0800079

[CIT0064] Soltis DE, Buggs RJA, Doyle JJ, Soltis PS. 2010. What we still don’t know about polyploidy. Taxon 59, 1387–1403.

[CIT0065] Soltis PS, Liu X, Marchant DB, Visger CJ, Soltis DE. 2014. Polyploidy and novelty: Gottlieb’s legacy. Philosophical Transactions of the Royal Society B, Biological Sciences 369, 20130351. 10.1098/rstb.2013.0351 PMC4071524 24958924

[CIT0066] Spoelhof JP, Soltis PS, Soltis DE. 2017. Pure polyploidy: closing the gaps in autopolyploid research. Journal of Systematics and Evolution 55, 340–352.

[CIT0067] Stebbins GL . 1971. Chromosomal evolution in higher plants. London: Edward Arnold Ltd.

[CIT0068] Strasser RJ, Tsimilli-Michael M, Qiang S, Goltsev V. 2010. Simultaneous *in vivo* recording of prompt and delayed fluorescence and 820-nm reflection changes during drying and after rehydration of the resurrection plant *Haberlea rhodopensis*. Biochimica et Biophysica Acta 1797, 1313–1326. 20226756 10.1016/j.bbabio.2010.03.008

[CIT0069] Strasser RJ, Tsimilli-Michael M, Srivastava A. 2004. Analysis of the chlorophyll *a* fluorescence transient In: Papageorgiou CG, Govindgee, eds. Chlrophyll fluorescence: a signature of photosynthesis. Dordrecht: Springer, 321–362.

[CIT0070] Stupar RM, Bhaskar PB, Yandell BS, et al. 2007. Phenotypic and transcriptomic changes associated with potato autopolyploidization. Genetics 176, 2055–2067. 17565939 10.1534/genetics.107.074286 PMC1950613

[CIT0071] Tank DC, Eastman JM, Pennell MW, Soltis PS, Soltis DE, Hinchliff CE, Brown JW, Sessa EB, Harmon LJ. 2015. Nested radiations and the pulse of angiosperm diversification: increased diversification rates often follow whole genome duplications. New Phytologist 207, 454–467. 10.1111/nph.13491 26053261

[CIT0072] te Beest M, Le Roux JJ, Richardson DM, Brysting AK, Suda J, Kubešová M, Pyšek P. 2012. The more the better? The role of polyploidy in facilitating plant invasions. Annals of Botany 109, 19–45. 22040744 10.1093/aob/mcr277 PMC3241594

[CIT0073] Thiebaut J, Kasha KJ. 1978. Modification of the colchicine technique for chromosome doubling of barley haploids. Canadian Journal of Genetics and Cytology 20, 513–521.

[CIT0074] Thompson JN, Merg KF. 2008. Evolution of polyploidy and the diversification of plant–pollinator interactions. Ecology 89, 2197–2206. 18724730 10.1890/07-1432.1

[CIT0075] Thompson KA, Husband BC, Maherali H. 2014. Climatic niche differences between diploid and tetraploid cytotypes of *Chamerion angustifolium* (Onagraceae). American Journal of Botany 101, 1868–1875. 25366852 10.3732/ajb.1400184

[CIT0076] Van Drunen WE, Husband BC. 2018. Immediate vs. evolutionary consequences of polyploidy on clonal reproduction in an autopolyploid plant. Annals of Botany 122, 195–205. 29726889 10.1093/aob/mcy071 PMC6025202

[CIT0077] Veselý P, Šmarda P, Bureš P, et al. 2020. Environmental pressures on stomatal size may drive plant genome size evolution: evidence from a natural experiment with Cape geophytes. Annals of Botany 126, 323–330. 32474609 10.1093/aob/mcaa095 PMC7380457

[CIT0078] Vyas P, Bisht MS, Miyazawa SI, Yano S, Noguchi K, Terashima I, Funayama-Noguchi S. 2007. Effects of polyploidy on photosynthetic properties and anatomy in leaves of *Phlox drummondii*. Functional Plant Biology 34, 673–682. 32689395 10.1071/FP07020

[CIT0079] Wang L, Luo Z, Wang L, Deng W, Wei H, Liu P, Liu M. 2019. Morphological, cytological and nutritional changes of autotetraploid compared to its diploid counterpart in Chinese jujube (*Ziziphus jujuba* Mill.). Scientia Horticulturae 249, 263–270.

[CIT0080] Wei N, Cronn R, Liston A, Ashman TL. 2019. Functional trait divergence and trait plasticity confer polyploid advantage in heterogeneous environments. New Phytologist 221, 2286–2297. 10.1111/nph.15508 PMC6587808 30281801

[CIT0081] Wei N, Du Z, Liston A, Ashman TL. 2020. Genome duplication effects on functional traits and fitness are genetic context and species dependent: studies of synthetic polyploid *Fragaria*. American Journal of Botany 107, 262–272. 31732972 10.1002/ajb2.1377

[CIT0082] Wendel JF . 2015. The wondrous cycles of polyploidy in plants. American Journal of Botany 102, 1753–1756. 26451037 10.3732/ajb.1500320

[CIT0083] Xing SH, Guo XB, Wang Q, Pan QF, Tian YS, Liu P, Zhao JY, Wang GF, Sun XF, Tang KX. 2011. Induction and flow cytometry identification of tetraploids from seed-derived explants through colchicine treatments in *Catharanthus roseus* (L.) G. Don. Journal of Biomedicine & Biotechnology 2011, 793198. 21660143 10.1155/2011/793198 PMC3110335

