# Supplementary material for: Phenotypic diploidization in plant functional traits uncovered by synthetic neopolyploids in Dianthus broteri
Source: J Exp Bot. 2021 Apr 28;72(15):5522–33. doi: 10.1093/jxb/erab179 (PMC8760854; doi:10.1093/jxb/erab179)
Supplement: erab179_suppl_Supplementary_Figures_S1-S2 [file erab179_suppl_supplementary_figures_s1-s2.pdf]

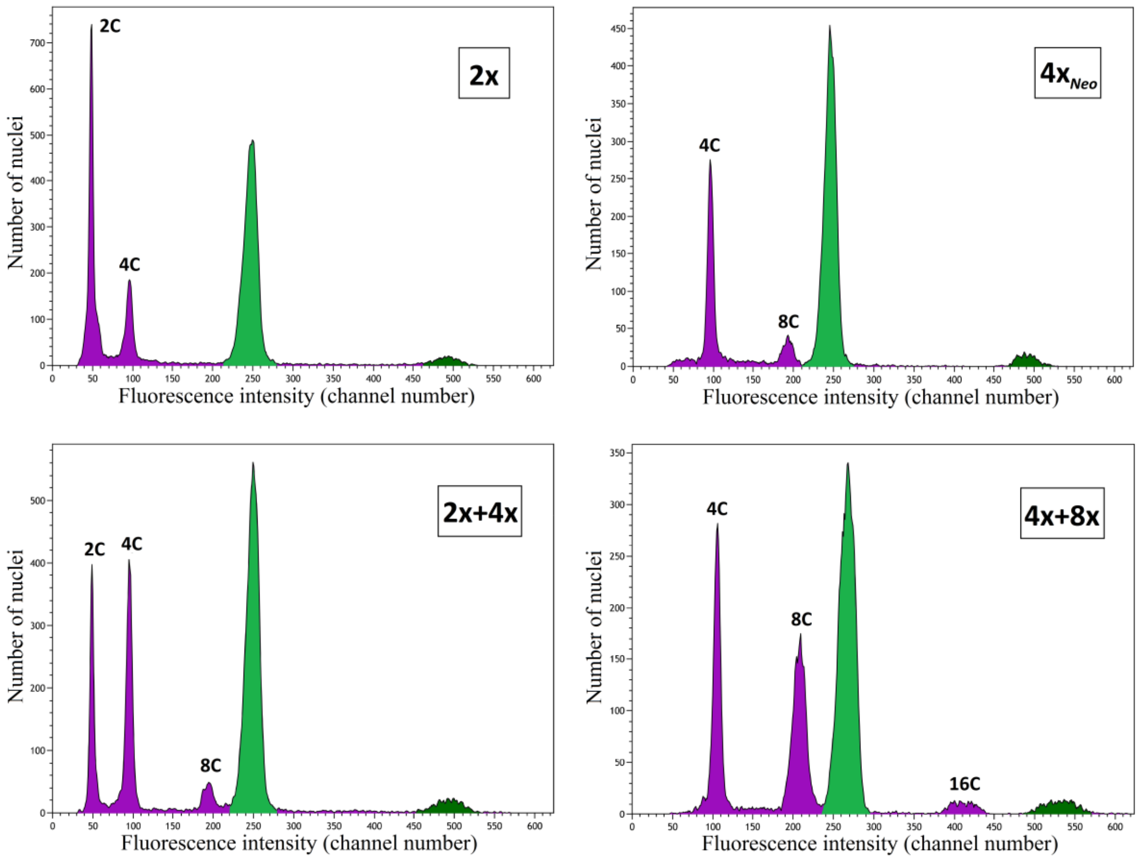

Fig. S1. Flow cytometry analyses of colchicine-treated plants. Each plant was classified as diploid ($2n$ = 2x), neotetraploid ($2n$ = $4x_{Neo}$), diploid-tetraploid mixoploids ($2n$ = 2x+4x) and tetraploid-octoploid mixoploids ($2n$ = 4x+8x) in base to their fluorescence peaks (marked in purple). Each purple peak is labelled in function of their DNA amount, being "C" the expected genome size of the *Dianthus broteri* haploid chromosome complement (~0.9 pg). Green peaks were derived from nuclei of *Pisum sativum* L. cv 'Ctirad', used as genome size control (2C nuclear DNA = 9.09 pg).

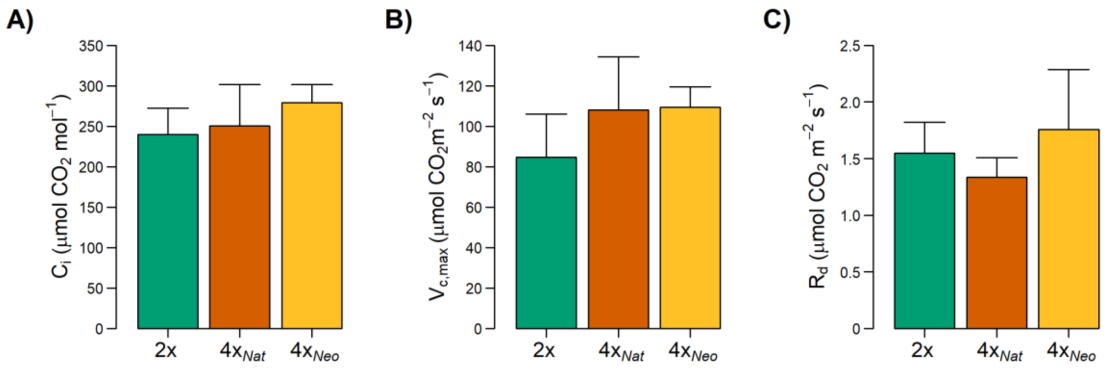

Fig. S2. Intercellular CO_2 concentration, C_i (A), maximum carboxylation rate allowed by ribulose-1,5-biphosphate (RuBP) carboxylase/oxygenase, $V_{c,max}$ (B), and rate of mitochondrial respiration during photosynthesis, R_d (C), of 2x, 4x$_{Nat}$ and 4x$_{Neo}$ *Dianthus broteri* cytotypes.
